# Supplementary material for: Splice-Junction-Based Mapping of Alternative Isoforms in the Human Proteome
Source: Cell Rep. Author manuscript; Available in PMC 2020 Jan 15. (PMC6961840; doi:10.1016/j.celrep.2019.11.026)
Supplement: 2 [file NIHMS1546469-supplement-2.zip › DF1/PXD006675/VentricularSeptum/VentricularSeptum_33_TTN_ASNEYGSVSCTATLTVTEPPK.pdf]

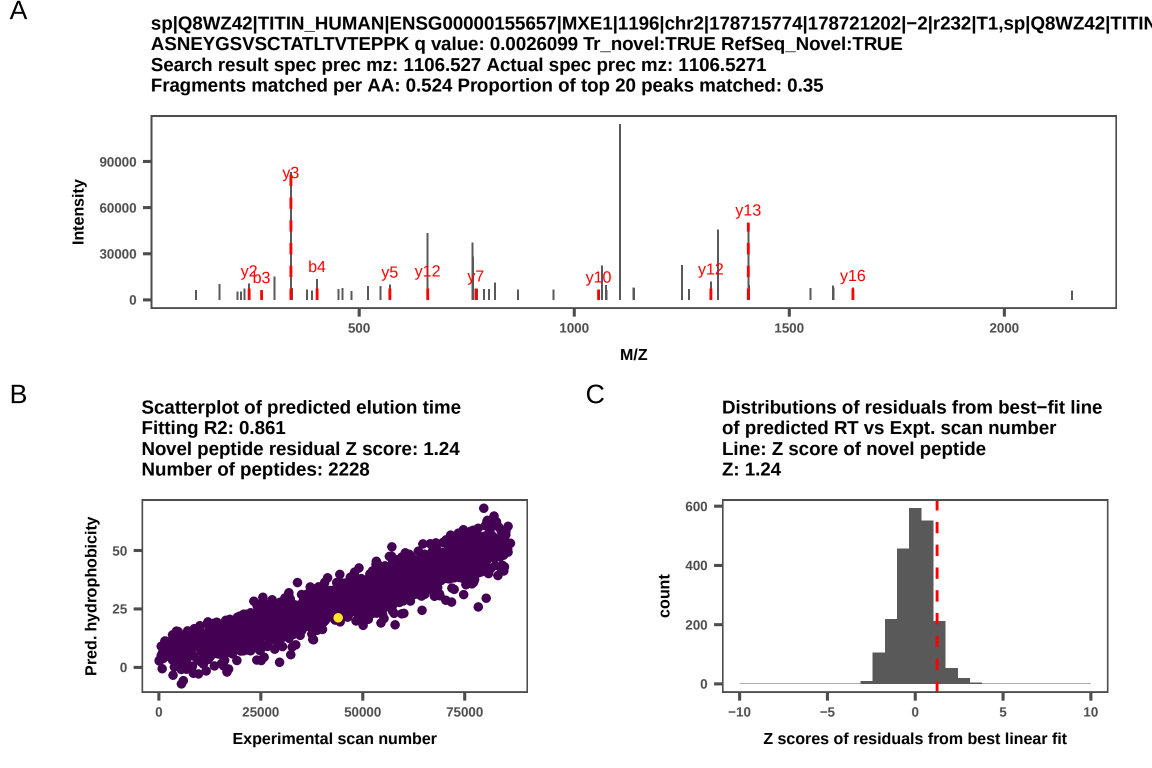

A
sp|Q8WZ42|TITIN_HUMAN|ENSG00000155657|MXE1|1196|chr2|178715774|178721202|−2|r232|T1,sp|Q8WZ42|TITIN
ASNEYGSVSCTATLTVTEPPK q value: 0.0026099 Tr_novel:TRUE RefSeq_Novel:TRUE
Search result spec prec mz: 1106.527 Actual spec prec mz: 1106.5271
Fragments matched per AA: 0.524 Proportion of top 20 peaks matched: 0.35
Intensity
90000
60000
30000
0
y3
y2
b3
b4
y5
y12
y7
y10
y12
y13
y16
500
1000
1500
2000
M/Z
B
Scatterplot of predicted elution time
Fitting R2: 0.861
Novel peptide residual Z score: 1.24
Number of peptides: 2228
Pred. hydrophobicity
50
25
0
0
25000
50000
75000
Experimental scan number
C
Distributions of residuals from best−fit line
of predicted RT vs Expt. scan number
Line: Z score of novel peptide
Z: 1.24
count
600
400
200
0
−10
−5
0
5
10
Z scores of residuals from best linear fit
